# Diffusive, Displacive Deformations and Local Phase Transformation Govern the Mechanics of Layered Crystals: The Case Study of Tobermorite

**DOI:** 10.1038/s41598-017-05115-4

**Published:** 2017-07-19

**Authors:** Lei Tao, Rouzbeh Shahsavari

**Affiliations:** 1 Department of Civil and Environmental Engineering, Rice University, Houston, TX 77005 USA; 2 Department of Materials Science and NanoEngineering, Rice University, Houston, TX 77005 USA; 3 Smalley Institute for Nanoscale Science and Technology, Rice University, Houston, TX 77005 USA

## Abstract

Understanding the deformation mechanisms underlying the mechanical behavior of materials is the key to fundamental and engineering advances in materials' performance. Herein, we focus on crystalline calcium-silicate-hydrates (C-S-H) as a model system with applications in cementitious materials, bone-tissue engineering, drug delivery and refractory materials, and use molecular dynamics simulation to investigate its loading geometry dependent mechanical properties. By comparing various conventional (e.g. shear, compression and tension) and nano-indentation loading geometries, our findings demonstrate that the former loading leads to size-independent mechanical properties while the latter results in size-dependent mechanical properties at the nanometer scales. We found three key mechanisms govern the deformation and thus mechanics of the layered C-S-H: diffusive-controlled and displacive-controlled deformation mechanisms, and strain gradient with local phase transformations. Together, these elaborately classified mechanisms provide deep fundamental understanding and new insights on the relationship between the macro-scale mechanical properties and underlying molecular deformations, providing new opportunities to control and tune the mechanics of layered crystals and other complex materials such as glassy C-S-H, natural composite structures, and manmade laminated structures.

## Introduction

## Size-dependent mechanical properties

The size-dependence of the strength of materials has attracted a great deal of attention in the past few decades. With the well-established dislocation and crystallography theory, different body-centered cubic (BCC), face-centered cubic (FCC), and hexagonal close-packed (HCP) nanomaterials have been investigated by both experimental and simulations approaches^1–9^. Although “smaller is stronger” is a commonly accepted paradigm, the underlying origin is still not thoroughly explored. Deformation mechanisms like twinning, martensitic transformation, and fracture, etc. are proposed to explain a material’s mechanical behavior, and among which dislocations are thought to have the greatest influence on the strength and deformation of a material^10–12^. Dislocation nucleation that is related to the incipient plasticity and strength limit has been observed in colloidal crystal^13, 14^. Theoretical studies are also conducted to describe and quantify the homogeneously nucleated defects^15^. Dislocation exhaustion contributes to Au pillars achieving the strength of about 800 MPa when the diameter of the pillar is about 300 nm^16–18^. The dislocation-interface interaction gives nano-twinned copper an uncommon combination of ultra-high yield strength of 1 GPa and high ductility of 14% ultimate elongation^19^. The ultra-strength is considered not only from the intrinsic feature of a material such as grain or precipitate size, twin boundary spacing, or dislocation density^20^, but also from the geometry of the loading applied to the material. Fleck *et al*.^21^ developed a plasticity law which was able to predict size-effects by postulating that the yield stress depended both on strain and strain gradient. Many subsequent studies explained the size-dependent mechanical properties successfully using the strain gradient plasticity (SGP) theory^22, 23^. The size effects can be rationalized especially under the circumstances of nano-indentation with the concept of underlying densities of statistically stored dislocations (SSD) and geometrically necessary dislocation (GND) contents^24, 25^. Analytic model based on GND is established to quantify the nano-indentation size-effect^26, 27^. For such dislocation-oriented structure, a gradual transition from size-independent to size-dependent behavior is discovered under nano-indentation, with size-effect emerging below a characteristic length scale^28^. The shape of indenter was also found to have different effect on the size-effect. For a pyramidal indenter, the measured hardness is increased with the penetration depth; while for a spherical indenter, the sphere radius is also an important factor besides depth^29^. Further study showed that the size-effect is manifest at depths less than 1 μm for pyramidal indenter, and with diameters less than 100 μm for spherical indenters^28^. Besides crystals, much attention are also put on promising materials like graphene and carbon nanotubes for their size dependent mechanical properties^30, 31^. Cement, however, as the key ingredient to concrete – the most widely used material in the world – has gained no attention on its size-dependent properties due to geometries of loading at nano scale; although it’s size effect has been observed and studied for many year at macro or micro scale^32–36^. The multi-scale diversity feature of cement presents many difficulties in studying its properties. As a starting point, here focus on calcium-silicate-hydrates (C-S-H) crystals, the major products of the cement hydration described in the next section. The current simulation is able to reach a scale of tens of nanometer, which is the dimension to be investigated for the size-effect of cement in our study. Although the absence of dislocation in C-S-H at such scale differentiates its size-effect significantly from conventional situations, the strain gradient mechanism still provides an explanation of the size-dependent properties. There is likely a transition from size-independent to size-dependent properties in C-S-H as the dimension approaches from hundreds of nanometers to few nanometers.

## C-S-H and its mineral analog tobermorite

C-S-H is the binder phase of concrete and its main source of the mechanical properties and durability^37, 38^. Its chemical composition and structure have been intensely studied for many years^39–47^. Pellenq *et al*.^43^ proposed a C-S-H molecular model based on information from experimental measurements. Their model is able to predict C-S-H’s essential structural features and mechanical properties consistent with experiment results. However, due to the C-S-H’s structural disorder and complexity, its mineral analog, tobermorite, is always served as a simple alternative to investigate^48, 49^. As for C-S-H’s mechanical properties and the underlying deformation mechanisms, Hou *et al*.^50^ employed uniaxial tension testing of C-S-H with different Ca/Si ratios using molecular dynamics by reactive force field and investigated their dynamics and mechanical properties. They revealed that the stiffness of C-S-H gel is weakened due to the presence of water molecules and the breakage of silicate chains. Manzano, *et al*.^51, 52^ calculated elastic properties of crystalline tobermorite and glassy C-S-H under shear using reactive force field simulations. They found that shear strain localizes at water-rich regions and the water controls their mechanical properties. Masoero *et al*.^53, 54^ explained the mechanical properties of C-S-H gels from nano-indentation experiments with a new proposed colloidal model. Their model offers a new approach to investigate the nano-indentation property of C-S-H gels as it simplifies the complex nanoporous structure of C-S-H into a system of spherical particles packed together. These works made significant advances on the mechanical properties and deformation mechanisms of C-S-H, but the deficiencies are also remarkable: (a) they studied the mechanical properties of C-S-H under different loading geometries for uniaxial tension, shear or nano-indentation, and there are currently no report on studying all loading geometries in a systematic and consistent manner; (b) their simulation works utilized reactive force field instead of C-S-H force field which is known to offer better prediction on mechanical properties of C-S-H and tobermorite^55^; (c) the indentation modulus is obtained not directly based on the nano-indentation simulation result, but based on elastic constants from the axial and shear simulation results^53, 54^. Furthermore, the experimental study of nano-indentation is on a scale of 300~500 nm^56, 57^, a very large scale unachievable by MD simulations, thus the comparison between experiments and simulation is unreliable. In order to overcome these deficiencies and provide a better alternative simulation strategy, the present work carried out a comprehensive study on the mechanical properties of layered tobermorite structure with axial, shear, and nano-indentation simulations, using a more proper C-S-H force field to reveal its loading geometry dependent mechanical behaviors.

The paper is organized as follows: First, the conventional loading geometries including tension, compression and shear are applied to the layered tobermorite structure, finding no size-dependent strength as macroscopic continuum theories still suffice. Then, nano-indentation is used as a loading geometry dependent experiment, which introduces strain gradients, demonstrating clear size dependent mechanical properties where the constitutive laws require length parameters. Finally, discussions and conclusions are provided.

## Simulation models

### The simulation model for conventional loading geometries

**Figure 1:**
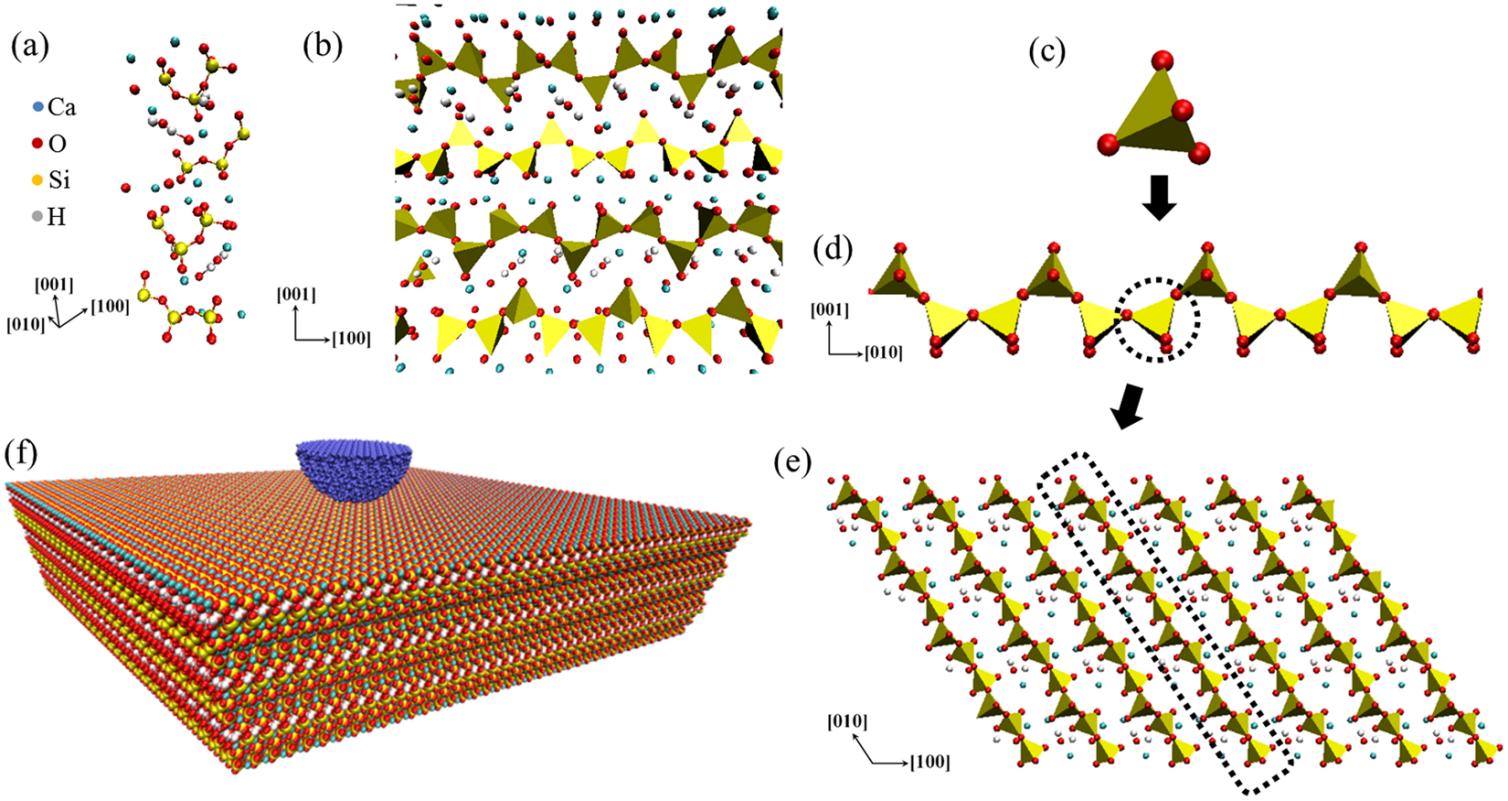
Atomic structure of 11 Å tobermorite investigated in this work. It shows a clear layered structure with backbone of Silicon-Oxygen tetrahedron. (**a**) the unit cell of tobermorite. (**b**) xz plane of tobermorite supercell. (**c**) Silicon-Oxygen tetrahedron. (**d**) Tetrahedron chain along [010]. (**e**) Tetrahedron layer along z axis. (**f**) Nano-indentation model of tobermorite substrate and an imaginary indenter.

The tobermorite is one of the crystalline minerals that are similar in composition to C-S-H. High resolution transmission electron microscopy (TEM) observations show that C-S-H gels contain tobermorite-like structure^58–61^. Tobermorite groups are layered structure and can be classified based on their different basal spacing as 9.3 Å, 11.3 Å and 14 Å which are usually referred to as 9 Å, 11 Å and 14 Å^62^. Figure 1 shows the atomic structure of 11 Å tobermorite^63^ investigated in this work.

The unit cell of 11 Å tobermorite (Ca_6_Si_6_O_14_(OH)_4_.2H_2_O with Ca/Si = 1.0) contains 72 atoms and is shown in Fig. 1a. It has a monoclinic structure and the lattice parameters are: a = 6.69 Å, b = 7.39 Å, c = 22.779 Å and γ = 123.49° ^63^. The simulation model 1 is constructed with unit cell into a 10 × 7 × 3 super cell for conventional loading geometries including tension, compression and shear. The final dimension is 66.9 Å × 51.73 Å × 68.337 Å with 15120 atoms in total.

The simulations performed in this paper utilized the molecular dynamics program LAMMPS (Plimpton, 1995), and CSH-FF force filed^55^, which is highly successful in predicting the mechanical properties of hydrated calcium-silicate materials^64, 65^. For each global deformation type, the molecular dynamics simulation is carried out in the isobaric-isothermal ensemble (NPT) for 5 picoseconds at 1 K, The integration time step is 0.1 femtosecond. Then the resultant configuration would be deformed via tension, compression and shear loading. Periodic boundary conditions are applied along three directions. All the deformations are applied in a quasi-static manner where every time the configuration is deformed a strain of 0.5%, then it is relaxed in canonical ensemble (NVT) for 1 picosecond. This quasi-static strategy is employed as it eliminates the strain rate effect on the mechanical properties to the greatest extent, allowing us to focus on the loading geometry dependent mechanical behaviors of the system.

### The simulation model for nano-indentation

The nano-indentation technology was developed 40 years ago to measure the hardness of materials^66^. Although by experimental means some properties of a material can be measured, the evolvement of the phase transformation at atomic level is hard to monitor. Molecular dynamics simulation of nano-indentation offers a new angle to get a comprehensive understanding of a material. The simulation model 2 for nano-indentation is constructed as a 22 × 24 × 6 super cell with the same unit cell used in global deformation. The final dimension of model 2 is 147.25 Å × 148.24 Å × 147.116 Å with 228096 atoms in total. Figure 1f shows a schematic diagram of the nano-indentation model used in the study. The tobermorite super cell is the substrate into which an imaginary rigid indenter shown in blue will be imposed.

The nano-indentation simulation is carried out in the canonical ensemble (NVT) at 1 K, and the integration time step is 0.5 femtosecond. The simulated system consists of a tobermorite super cell substrate and an imaginary sphere indenter with the radius of the following four values: 20, 30, 40, and 50 Å. The indentation is applied along the negative direction of the x, y and z axis at a constant rate of 0.1 Å/ps (10 m/s), and periodic boundary conditions are applied along the directions perpendicular to the indent direction. To avoid the rigid body movement of the whole substrate, the bottom silica chain of the substrate is kept fixed throughout the simulation. In all these three situations, the indenter moves into the substrate from the surface of the structure. Since it’s more convenient to carry out indentation simulation with constant rate loading, quasi-static strategy is not employed here, but the effect of the loading rate will be discussed later. A proper rate is chosen so that the strain rate is not a concern and only loading geometry dominates the mechanical properties.

## Results and Discussion

When it comes to loading complex and homogenous crystals such as tobermorite, understanding the type and nature of their deformation mechanisms (e.g., whether there are dislocation-like mechanisms) is the key to mechanical properties. We found that not only there exists a dislocation-like ‘displacive mechanism’ in tobermorite, but there are ‘diffusive mechanisms’ and ‘local phase transformation’ that dominate the behavior of tobermorite on certain conditions. Displacive deformation mechanisms imply that atoms move collectively to the new equilibrium position, while diffusive deformation mechanisms indicate that atoms move chaotically to the new equilibrium position^10^. Herein we classified the deformation type of tobermorite into two categories: global and local deformations in order to characterize the underlying deformation mechanisms in this layered material. Global deformations includes conventional loading geometries like tension, compression and shear which bring no size-dependent strength, while local deformation related to nano-indentation is another loading geometry, which introduces size-dependent mechanical behavior.

### Global deformations due to conventional loading geometries

**Figure 2:**
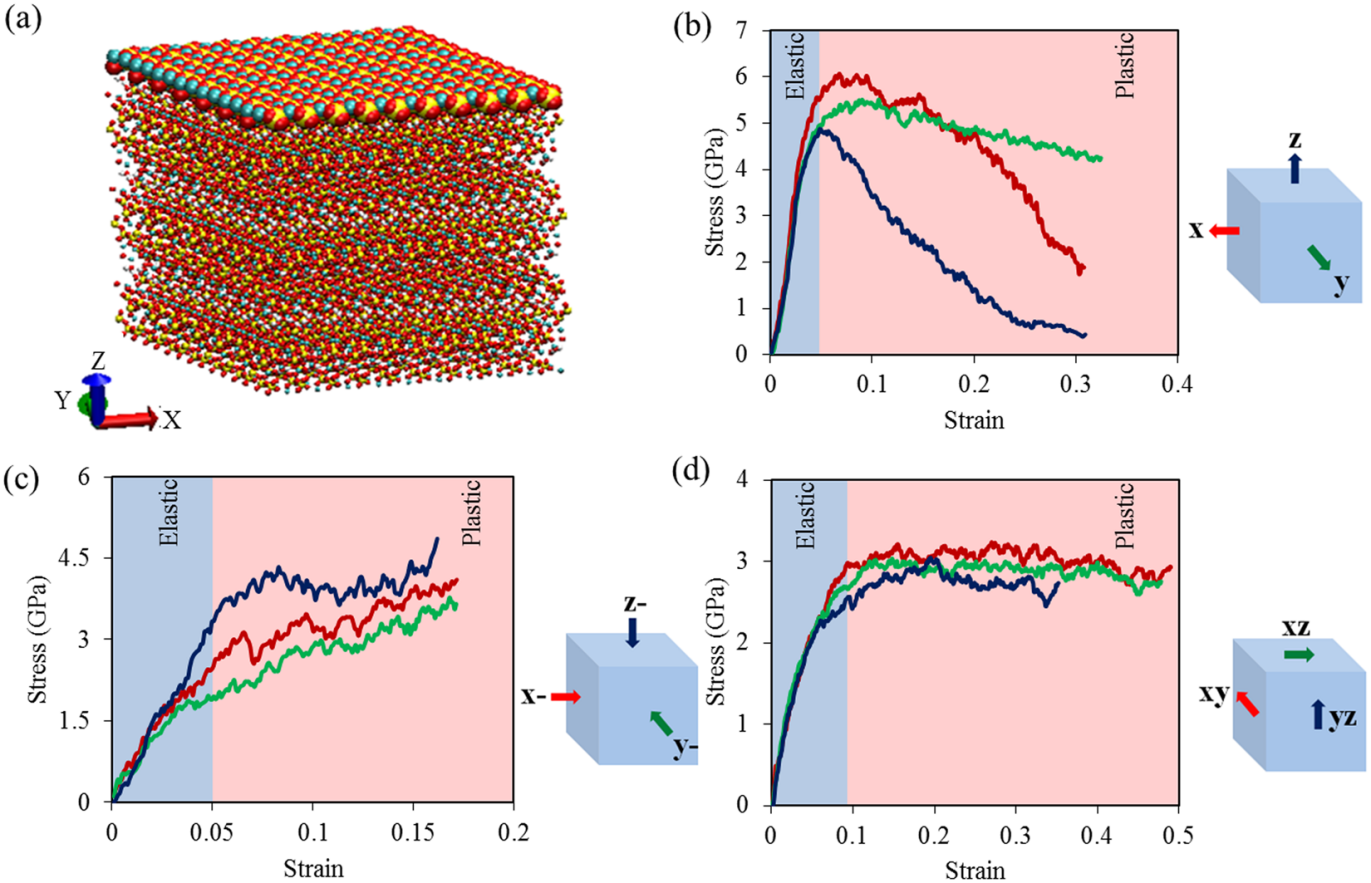
(**a**) The tobermerite super cell highlighting the top calcium-silicate layer. (**b**) Stress-Strain curves for tension along x, y, and z axis, which are illustrated by the red, green, and blue curves, respectively. (**c**) Stress-Strain curves for compression along x, y, and z, which are illustrated by the red, green, and blue curves, respectively. (**d**) Stress-Strain curves for shear in xy, xz and yz plane, which are illustrated by the red, green, and blue curves, respectively. The blue region is the elastic stage, while the red region is the plastic-damage stage.

The tobermorite super cell of model 1 is shown in Fig. 2a in which x, y, and z indicate the directions of the Cartesian coordinates. Since the unit cell of tobermorite is monoclinic, the crystallographic orientation [010] does not coincide with the y axis. It’s worth noticing that the deformation is always applied in terms of Cartesian coordinates. In the discussion below, the tension along x, y, and z axis are referred to as “x”, “y”, “z”; the compression along x, y, and z axis are referred to as “x-”, “y-”, “z-”; and the shear in xy, xz and yz plane are referred to as “xy”, “xz”, “yz”.

Figure 2a shows the tobermorite supercell highlighting the top layer. Owing to the characteristic that the layered stacking along z axis are the principle representatives of the tobermorite, our focus is placed onto the behavior of the layers. Their geometric feature and atomistic configuration are investigated when the structure is deformed in different directions. The backbone of the tobermorite can be divided into three levels from small to big: 1. Silicon-Oxygen tetrahedron →2. Tetrahedron chain along [010] → 3. Tetrahedron layer along z axis (see Fig. 1c–e).

In the plots of the stress-strain curves in Fig. 2, the blue region and red region mark the elastic stage and the plastic-damage stage of the each deformation. The elastic modulus can be obtained from the slope of the linear portion of stress-strain curves. For deformation type “x”, “y”, “z”, “x-”, “y-”, “z-”, “xy”, “xz”, and “yz”, the elastic modulus are 99.17 GPa, 98.07 GPa, 96.18 GPa, 62.08 GPa, 47.94 GPa, 65.72 GPa, 60.17 GPa, 67.38 GPa, 70.95 GPa, respectively. After averaging all the elastic constants from each simulation, Table 1 shows that the elastic constants calculated by molecular dynamics simulation agree well with the ones from first-principles calculation^67, 68^, making our MD simulation results reliable.

**Table 1:** Comparison of elastic constants (GPa) of tobermorite (The bold values are for elastic modulus along 6 main directions).

	First principle	Molecular dynamics		First principle	Molecular dynamics		First principle	Molecular dynamics
**C11**	**102.65**	**105.92**	C23	18.83	56.73	C36	−3.38	−12.21
C12	41.68	43.77	C24	0.00	9.48	**C44**	**22.90**	**59.34**
C13	27.70	36.36	C25	0.00	−0.92	C45	−11.93	−1.28
C14	0.00	25.91	C26	−4.10	−0.61	C46	0.00	−0.91
C15	0.00	−13.78	**C33**	**83.80**	**82.82**	**C55**	**23.25**	**67.30**
C16	1.25	−3.91	C34	0.00	19.35	C56	0.00	3.28
**C22**	**125.05**	**136.00**	C35	0.00	−29.26	**C66**	**50.20**	**55.65**

For an identical deformation type (e.g. tension), the structure would behave differently along different directions, which is an indication of structural anisotropy. For example, considering the compression results shown in Fig. 2c along “z-” condition, as the compression proceeds, the stress increases linearly in the elastic range until a strain of 0.07 and the stress reaches 4 GPa. After the elastic range, the stress enters into a plateau, displaying a good plasticity and giving a sign of drastic structural rearrangement for the layered configuration.

Compared to “z-”, in the “x-” and “y-” conditions, the stress curves show a bi-linear character: the stress first increases linearly to a strain of 0.07 in the elastic range, and subsequently increase linearly with a smaller slope to enter into plastic regime slowly, but the stress level is smaller than that of “z-”. The difference between “x-”, “y-” and “z-” is because “x-” or “y-” leads to the damage of the planes, while “z-” prefers to maintain the integrity of each layer, exhibiting a high loading capacity and a significant compatibility in plastic deformations.

Regarding tension results in Fig. 2b, all of the stress curves increase linearly initially at a strain range of 0 to 0.05, and at the end of elastic stage, the maximum stresses are reached for “x”, “y”, “z” at 5.93 GPa, 5.20 GPa, and 4.87 GPa, respectively. Following a plastic plateau from a strain of 0.05 to 0.1, the stress curves for “x”, “y” decrease with different rates. However, stress curves for “z” turned downwards immediately after the ultimate value. The rapid decrease for “z” indicates the splitting of neighboring layers requires only a small load, due to the weak hydrogen bond connections between the tetrahedra in adjacent sheets and the water molecules. Hydrogen bond connectivity is known to be one of the key factors in cohesion between calcium-silicate-hydrate layers and is important for the mechanical behavior^69^.

The decreasing rates of stress curves for “x” and “y” are higher than “z”, since the damage of the planes means the breakage of tetrahedron chains. Strong Ca-O bond and Si-O bond contribute to the high strength of the silicate chains^68^. The silicate chains are aligned in the direction [010] which is closer to the “y” direction; therefore the decreasing rate of stress curves for “y” is smaller than that “x”.

Considering shear results in Fig. 2d, the stress curves can be divided into two sections: the first elastic regime between the strain of 0 and 0.1, and the second regime of plastic plateau subsequently. The stress is lower when shear is applied along “xz” and “yz”. These two shear deformations cause relative gliding between neighboring layers connected mainly by weak H-bond connections. This explains why smaller loads are needed compared to shearing the layers along “xy”.

Obviously, all the stress-strain curves are good reflections of the structures’ layered feature. On one hand, within one layer, strong Ca-O and Si-O bonds contribute to good intra-plane mechanical properties. On the other hand, weak H-bond connectivity between layers contributes to poor inter-plane mechanical properties. The intra- and inter-plane effects are interactive to influence the overall mechanical properties. When the H-bond is vulnerable under specific deformation like shear, the maximum stress is only around 3 GPa. As long as Ca-O and Si-O bonds undertake most of external influences like tension along the “x” axis, the maximum stress increases to around 6 GPa. From an atomistic perspective, these mechanical properties are predicted and explained satisfactorily, indicating that atomistic configurations are key factors in determining the material’s properties.

The atomic configurations of the structure at strain of 0 and stain of 0.1 are compared below to explain the deformation mechanisms in the context of global deformation. After the strain reaches 0.1, the structure behaves plastically for all deformation types. Despite the detail of the classified displacive controlled and diffusive controlled deformation mechanisms, the good agreement of the calculated elastic constants in Table 1 shows the absence of size-dependent mechanical properties, since the ab-initio data are performed in a much smaller size (unit cell). The strain in the system is uniform with a large length scale of the deformation field. The small strain gradient in global deformation type is believed to be the reason behind size-independent properties.

**Figure 3:**
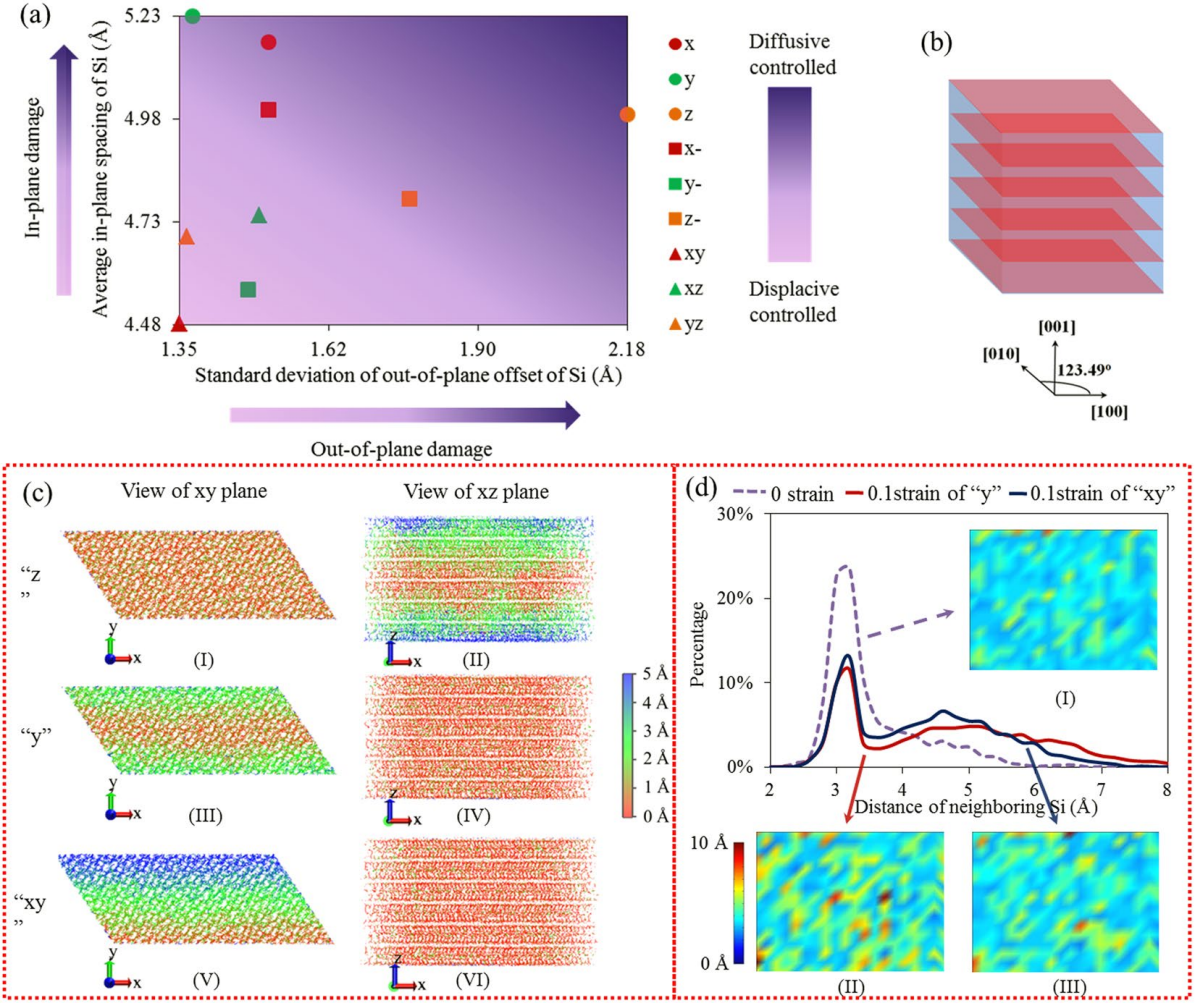
(**a**) Deformation Mechanism Map showing the deformation mechanism corresponding to different global deformation types. The in-plane and out-of-plane damage indexes are calculated when the strain reaches 0.1. (**b**) Schematic diagram of layers stacking along z axis in tobermorite. (**c**) Displacement Map: (I) xy plane view of tension along z axis, (II) xz plane view of tension along z axis, (III) xy plane view of tension along y axis, (IV) xz plane view of tension along y axis, (V) xy plane view of shear in xy plane, (VI) xz plane view of shear in xy plane. (**d**) Distribution of Distance between neighboring Silicon atoms: (I) Distance Map for neighbor Silicon atoms at strain of 0, (II) Distance Map for neighbor Silicon atoms with tension along y axis at strain of 0.1, (III) Distance Map for neighbor Silicon atoms with shear in xy plane at strain of 0.1.

At atomic level, we classify all the deformation mechanisms into displacive and diffusive mechanisms for all the global deformation types (tension, compression, shear). With displacive mechanisms, atoms behave in a more collective and deterministic manner like the dislocation glide in crystal structures. However, in diffusive mechanisms the atoms behave in a more random manner^10^. These two mechanisms can be determined by characterizing the out-of-plane damage and in-plane damage of each layer. To describe the out-of-plane damage of each layer, z coordinates of silicon atoms within one layer are averaged first as the position of the layer, then the standard deviation of such coordinates is calculated to describe the integrity of the layer. The larger the standard deviation, the more the out-of-plane damage. In order to describe the in-plane damage of each layer, the distances between neighboring silicon atoms are averaged as an index to describe the in-plane damage. The larger the sum of the distances, the more the in-plane damage. Figure 3a shows all the in-plane and out-of-plane damages of all the global deformation types, differentiating their deformation mechanisms. The abscissa of Fig. 3a indicates the out-of-plane damage index and the ordinate indicates the in-plane damage index.

The pinkish color in the left corner of Fig. 3a gives a region with relatively small abscissa and ordinate value, indicating displacive-controlled deformation mechanism, while the purple-like color on the top right region is regarded as diffusive-controlled deformation mechanism. All the shear deformations (“xy”, “xz”, “yz” shown as triangle) belong to displacive controlled deformation while all the tension (shown as circles) and compression (shown as squares) deformations belong to diffusive-controlled deformation except “y-” which belongs to displacive-controlled deformation (to be discussed shortly).

To further clarify the above mechanisms, the change in the model’s atomic configuration is presented with displacement maps. In the study of perfect crystal structures, displacement map is constructed to analyze potential mobility of the dislocations^70^. The displacement map employed in this study is a colored map showing the displacement of each atom before and after the deformation. Figure 3c shows the displacement maps for “z”, “y”, and “xy” deformation modes, respectively. According to Fig. 3c(II), the displacement map in the z direction shows that there is no clear boundary between different colors, meaning that all the atoms deform individually, which give a large abscissa value to the “z” point in Fig. 3a. On the contrary, from Fig. 3c(IV) and Fig. 3c(VI), the almost uniform color shows that there is no diffusion in z direction (out of plane damage), resulting in similar small abscissa values to “y” and “xy” points in Fig. 3a.

Figure 3c(V) shows a clear boundary between different colors, meaning that all the atoms behave collectively to deform, so the shear deformation in the xy plane can be treated as dispacive-controlled deformation (The “xy” point in Fig. 3a locates in the pink region with a small ordinate value). Although Fig. 3c(IV) shows a similar pattern as Fig. 3c(VI), its deformation mechanism is different because the “y” point in Fig. 3a has a larger ordinate value than “xy” point. In other words, the in-plane damage of deformation type “y” is more serious than that of “xy”. This more serious in-plane damage makes “y” belong to diffusive-controlled deformation mechanism. Indeed, it is easier to explain the difference between “y” and “xy” via Fig. 3d, which shows the distribution of distance between neighboring silicon atoms. Before deformation, the distance between neighboring silicon atoms is around 3.4 Å. After different deformation types with strain of 0.1, a second distribution peak occurs. This second peak is an indicative of the in-plane damage. It can be seen that the curve of “y” is more uniform, or its second peak is blunter than that of “xy”. For deformation type “y”, the distance between neighboring silicon atoms ranges from 3 to 7 Å on average. This feature also suggests that when the tension is along the y axis, the atoms deform individually along the in-plane direction.

Figure 3d(I–III) are the distance map for neighboring silicon atoms in the middle layer, the sky blue is related to a distance of ~3.4 Å and the yellow and red are signs of in-plane damage. Comparison of Fig. 3d(II) and d(III) shows that the in-plane damage of “y” is more severe than that of “xy”, so for “xy”, the layer is kept almost intact and the deformation mechanism is displacive-controlled with atoms’ collectively moving to new positions.

Using similar analysis, other global deformation types are also categorized into either displacive- or diffusive-controlled deformation mechanisms in Fig. 3a. It is worth noting that the shear deformations “xy”, “xz”, and “yz” are dominated by displacive-controlled deformation mechanism; the tension deformations “x”, “y”, and “z” are dominated by diffusive-controlled deformation mechanism while the compression deformations fall in between, except “x-” and “z-” which are dominated by diffusive controlled deformation mechanism and “y-” by displacive-controlled deformation mechanism. Overall, the shear, compression, and tension deformations rank from high to low in terms of the extent to which the system can maintain its structural integrity.

### Local deformations due to nano-indentation

From our above simulation results, the calculated indentation modulus, *M*, of tobermorite is calculated with Reuss-Voigt-Hill approximation^71^ and Molinary approximation^72^ by

1 $$M=4G\frac{3K+G}{3K+4G}$$

where *K* and *G* are, respectively, the bulk and the shear moduli. Table 2 indicates that the indentation modulus from simulation agrees well with that from first-principles calculations^67^. Constantinides *et al*.^57^ carried out nano-indentation on C-S-H and gave statistical distribution of elastic modulus for C-S-H, the obtained elastic modulus for nondegraded C-S-H varied from 18 to 36 GPa. Because of the defects in the material, this smaller elastic modulus is expected as compared to our simulation results. It’s also worth noticing that the imprint of nano-indentation experiment carried by Constantinides *et al*. is several hundred nanometers. Their indentation depth varied in the range of 300–500 nm, while the maximum indentation depth in our simulation is 5 nm, two orders of magnitude less than the experiment. Qomi *et al*.^64^ calculated the C-S-H solid particle’s indentation modulus *M* using same formula (1) considering the effect of Ca/Si ratio. The value they obtained varies from 60 to 100 GPa, which is also in the same order of magnitude with Table 2. The calculated nano-indentation moduli mentioned above are actually from the elastic constants we obtained from axial and shear deformation simulations. As we discussed in section 3.1, all these defamation types are devoid of strain gradients and relate size-independent mechanical properties, so it’s not surprising that the derived nano-indentation moduli match well with the first principles calculations value and those of experimental studies. The actual indentation experiments^57^ are expected to obtain reliable mechanical properties such as elastic modulus and hardness, and there is no size-effect at such scale of 300–500 nm. However, around our simulation scale of 5 nm, size-dependent mechanical properties start to be manifested as discussed later. At micro scale, the behavior of materials may change on different dimensions ranging from few nanometers to hundreds of nanometers when different deformation mechanisms function along or in combination. Dislocation nucleation, dislocation motion, grain interaction, phase transformation, etc. are examples of the possible mechanisms responsible for size-effects on different length scales. There is a critical length above which size-independent properties can be measured and explained by classical theories. But the comprehensive investigation of size-effect up to the possible critical length of hundreds of nanometers is currently computational very expensive (if not impossible). Our investigated size-effect on the scale of 5 nm reveals part of the whole phenomenon.

**Table 2:** Comparison of indentation modulus [GPa].

	First-principles calculation	Molinary Approximation	Reuss-Voigt-Hill average of the elastic constants
K	52.68	57.41	56.92
G	29.81	27.73	42.02
M	80.77	78.33	105.55

**Figure 4:**
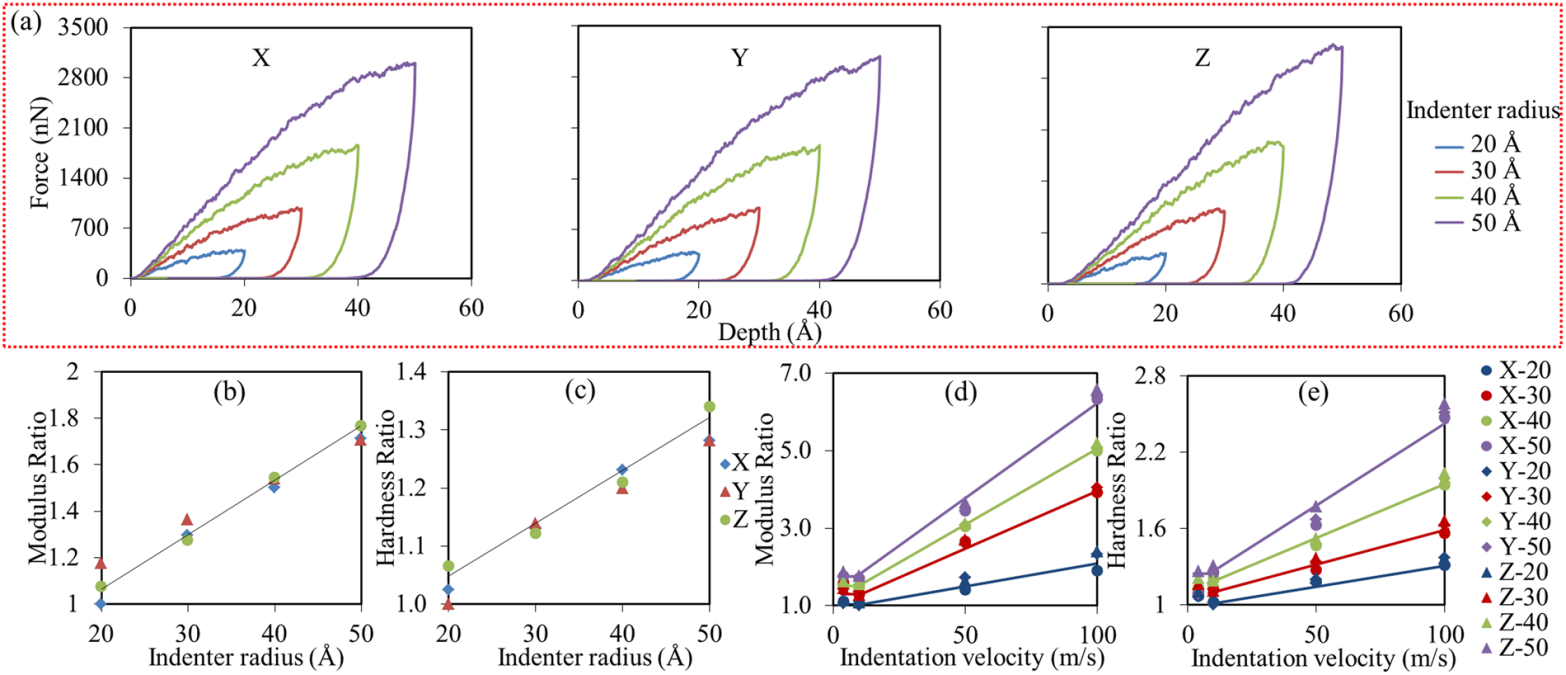
(**a**) Force-depth curves of nano-indentation. (**b**) Normalized indentation moduli versus indenter size (**c**) Normalized hardness versus indenter size; (**d**) Normalized indentation moduli versus loading rates (**e**) Normalized hardness versus loading rates. The normalization factor is the indentation modulus or hardness of the system with the smallest indenter size.

A totally different scenario happens when we look at the force-depth curves obtained from our nano-indentation simulation of model 2. We choose a spherical indenter with four different sizes to study the size-dependent characteristic of this specific geometry of loading. When the radius of indenter is chosen as 20, 30, 40, and 50 Å, the corresponding maximum indentation depth is set to be equal to the radius of the indenter (see the movies in the supplementary information). Figure 4a shows the force-depth curves of loading and unloading from the nano-indentation simulations along three Cartesian directions. We calculated the indentation modulus *M* and hardness via^73, 74^.

2 $$M=\frac{1}{2}\frac{dP}{dh}\frac{\sqrt{\pi }}{\sqrt{A}}\,\,H=\frac{{F}_{\max }}{A}$$

where *M* is the indentation modulus, *H* is the hardness, *dP/dh* is the slope of the elastic unloading, *A* is the area of contact, and *F*_max_ is maximum force applied. Interestingly, we found a linear pattern between the normalized indentation modulus and the radius of indenter, i.e. the larger the radius of the indenter, the larger the indentation modulus. A similar linear relationship, albeit with a different factor, exists for the normalized hardness. This size-effect can be explained by the concept of strain gradient.

When exploring the strain gradient origin in size-effect studies, other possible reasons such as loading rate should be excluded first. We compared the results with loading rate of 0.04 Å/ps (4 m/s), 0.1 Å/ps (10 m/s), 0.5 Å/ps (50 m/s), and 1 Å/ps (100 m/s) for indentation modulus and hardness in Fig. 4d,e. The trend indicates that the adopted loading rate 0.1 Å/ps (10 m/s) is small enough to not affect the mechanical properties of the structure, which is also verified in the study of other mineral materials^75^.

**Figure 5:**
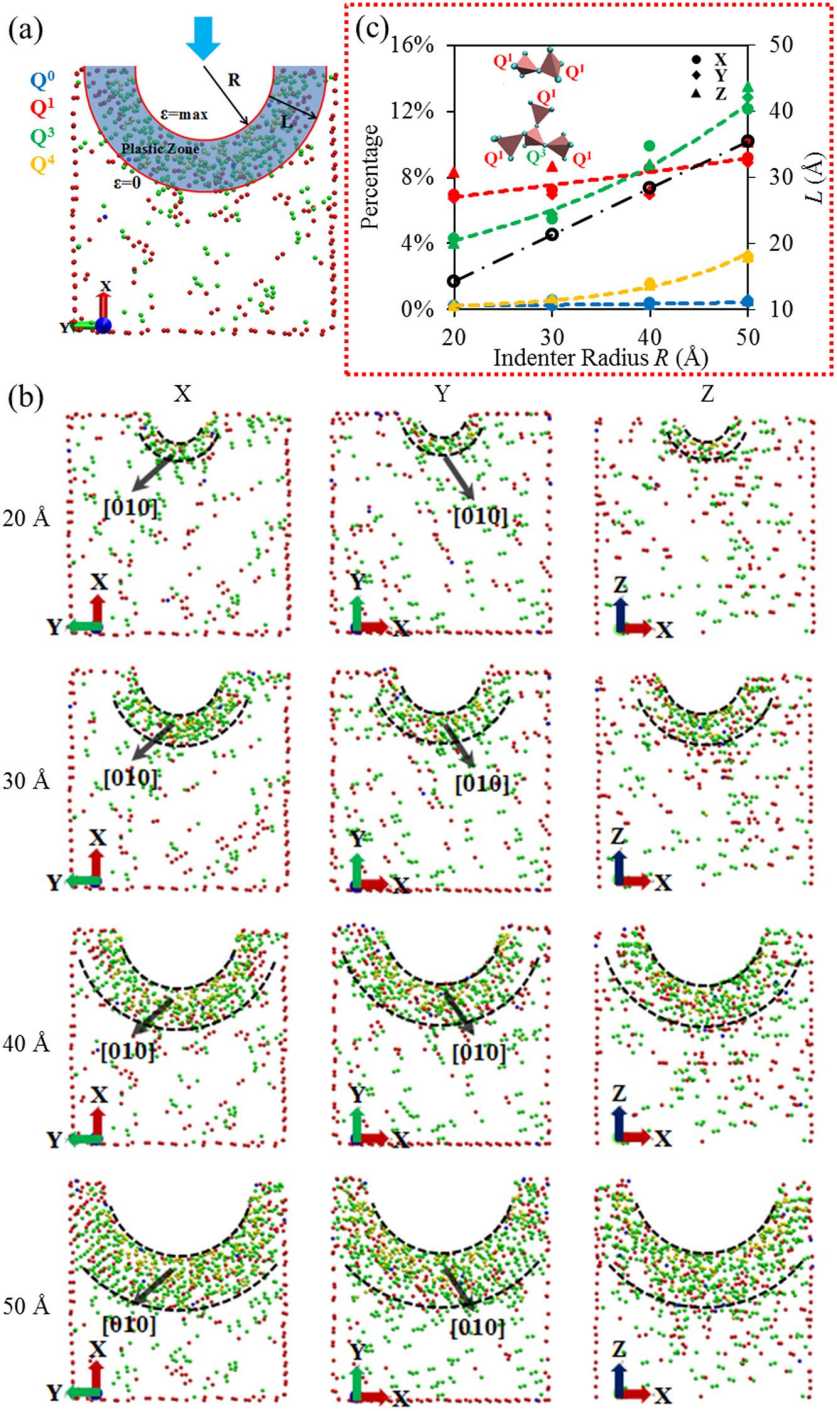
(**a**) Phase transformation in the plastic zone during nano-indentation with a 40 Å radius indenter depressing along X axis. (**b**) Side cross-sectional view of the indented structure with different indenter size depression along each direction. (**c**) The comparison of Q^0^, Q^1^, Q^3^, Q^4^ of the indented structure with different indenter size depression along each direction (colored curves corresponding to the left ordinate); and the linear relationship between *L* and *R* (black line corresponding to the right ordinate).

The indentation-induced size-effect observed in crystalline metallic materials is successfully explained by densities of geometrically necessary dislocation (GND) contents in the plastic zone underneath the indenter^25^. So it’s natural to check what’s happening in the plastic region of our model underneath the indenter. Because of the layered structure of tobermorite (unlike crystalline structures such as metals) and the small scale of our model (~10 nm), there is no dislocation observed in our system. But an underlying local phase transformation of the tobermorite substrate from crystalline to amorphous structure is monitored in the process of nano-indentation simulation. Akin to perfect crystal structures where atoms are differentiated by their coordination number as a sign of dislocations, here SiO_4_ tetrahedra are differentiated as the sign of local phase transformation in the nano-indentation simulations of tobermorite. As discussed earlier, the backbone of the tobermorite is composed of SiO_4_ tetrahedra. A tetrahedra with n bridging oxygen atoms is typically referred as Q^n^, a common notation in nuclear magnetic resonance (NMR) field for describing local connectivity of amorphous-type materials^76, 77^. Generally, Q° to Q^4^ represents the monomer, the dimer structure, the long chain, the branch structure, and the network structure, respectively^78^. In the pristine tobermorite crystal, Q^2^ is the only component, indicating infinite chains of SiO_4_. As the indenter enters into the tobermorite substrate, phase transformation happens at the local plastic region around the indenter, resulting in emergence of new Q^0^, Q^1^, Q^3^, and Q^4^. This phenomenon can be observed in the atomic configuration plots in Fig. 5a in which the side cross-sectional view of the indented structure in the case of indentation along x axis is shown (40 Å radius of indenter; see movie indentX in the SI). Our analyses show that new phases extend annularly into the tobermorite substrate with Q^4^ being in the innermost, indicating that the most dramatically phase transformation happens in close proximity to the indenter. When the indentation is applied along y axis (see movie indentY in the SI), the pattern of local phase transformation is similar with the former case. It is observed that as the indenter moves along x or y axis, the new phases extend along the [010] direction, i.e the direction of infinite Si tetrahedra chains (Fig. 5b). This extension along specific direction endows the local phase transformation a feature that the defects happen along a crystallographic direction. Inconspicuous pile-up around the indenter involves in all cases, although the pile-up is clearer when the indentation is along z axis (refer to movie indentZ in the SI). The little pile-up is believed to have little effect on the mechanical properties of the structure.

Figure 5b demonstrates the phase transformation for 12 different cases (4 indenter radii and 3 indentation directions) in which the local transformation happens at a plastic zone closest to the indenter, a region most dramatically affected by the indentation. Careful analysis of the plastic zone in Fig. 5a shows that the zone is actually a volume between two hemispheres, one with a radius of *R* and one with a radius of *R* + *L*. At the inner hemisphere surface with area 2*πR* ^2^, the strain ε is maximum, while beyond the outer hemisphere surface with area 2*π*(*R* + *L*)^2^, the strain ε is lowest. By investigating all 12 local phase transformation scenarios in Fig. 5b, we found that the strain gradient length *L* (along which the strain varies from maximum to zero) is proportional to the radius of the indenter *R*, i.e. *L*∝*R* (shown with black line in Fig. 5c). Furthermore, the representative length scale of the maximum strain *S* is proportional to the area of inner hemisphere surface 2*πR* ^2^, namely *S*∝*R* ^2^. So the strain gradient *SG* can be defined clearly as *SG* = *S/L*, thus we get *SG∝R* ^*2*^ */R*, or *SG∝R*. In view of our simulation result, equations (3) and (4), and the definition of strain gradient, equation (5) is concluded which explains the linear size-effect in Fig. 4b,c.

3 $$L\propto R$$

4 $$S\propto {R}^{2}$$

5 $$SG=S/L\propto R$$

Obviously, the strain gradient interpretation is drawn from the perspective of geometric scale of the plastic zone. The atomistic origin behind it is also clear. Figure 5c illustrates the number of Q^n^ corresponding to the configurations shown in Fig. 5b. The number of each transformed Q^n^ is increasing as the indenter’s radius increases; this pattern is responsible for the linear increase of the indentation modulus and hardness in Fig. 4b and c. The original tetrahedron chains are mainly changed into two atomic clusters containing Q^1^ and Q^3^. Along the z axis, the indentation leads to more Q^1^ and Q^3^, suggesting a more severe local phase transformation along this direction. Compared to others, Q^3^ is the most sensitive local connectivity and it is believed to be the main source of the increased indentation modulus. This is in line with the previous experimental studies that indicate Q^3^ species are able to improve the interlayer stiffness of tobermorite^79^.

**Figure 6:**
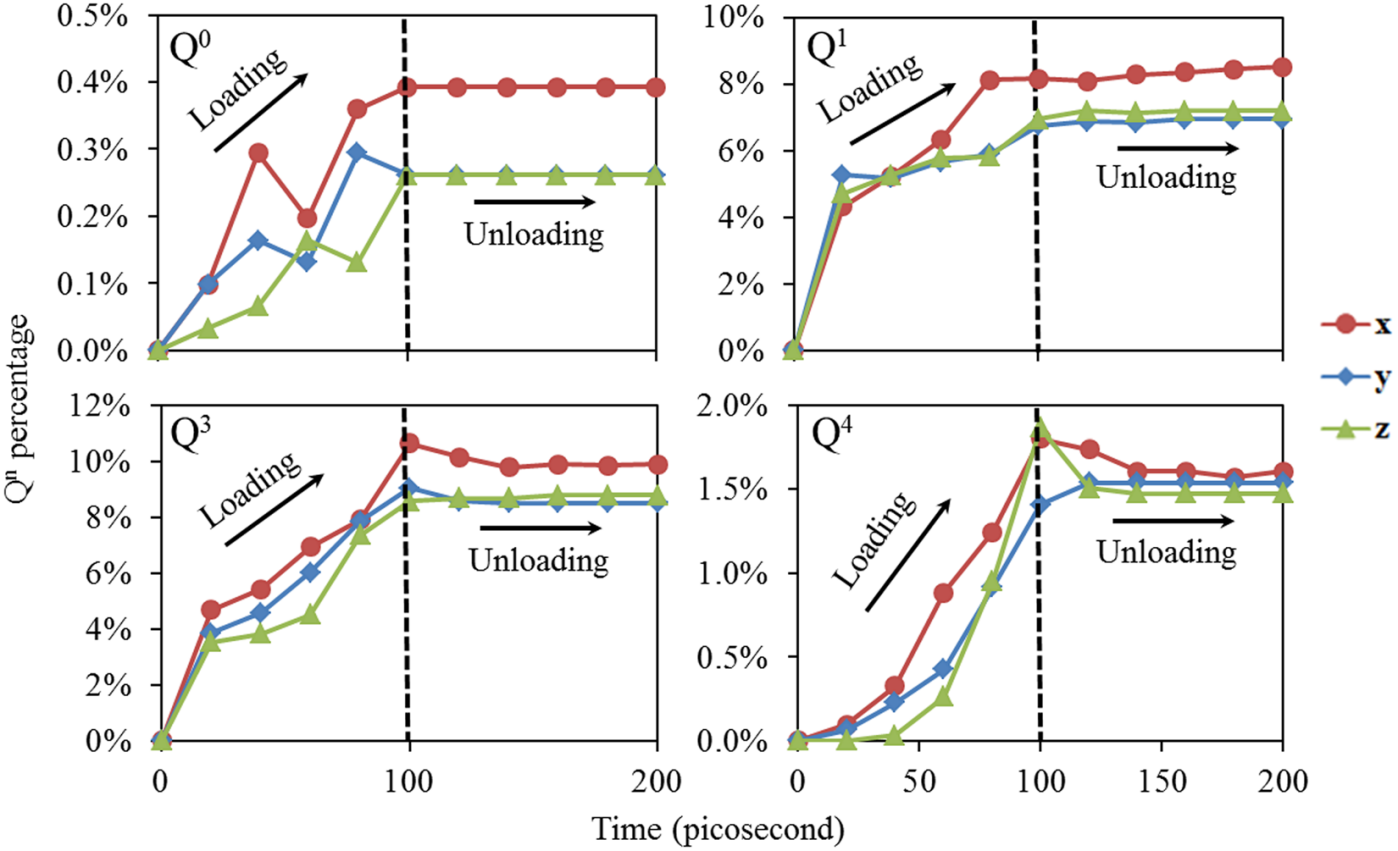
Time-dependent process of the phase transformations during nano-indentation with a 40 Å radius indenter.

Figure 6 demonstrate a typical time-dependent process of phase transformation for the indentation simulation with 40 Å as the radius of the indenter. The time regime of 0 to 100 ps represents loading where the indenter moves into the substrate while 100 to 200 ps corresponds to the unloading stage. Figure 6 illustrates that the number of Q^0^ and Q^1^ increases as the loading proceeds and then remains constant during unloading. A similar pattern exists for Q^3^ and Q^4^ except that at the beginning of the unloading stage, the number of Q^3^ and Q^4^ decreases slightly and then remains constant. Since Q^3^ and Q^4^ denote larger and more complex atomic cluster, it is not surprising to find that Q^3^ and Q^4^ are more sensitive to unloading. Once the indenter detaches completely form the substrate, the locally phase transformed structure don’t change any more, indicating permanent (plastic) deformations.

## Conclusion

This work investigated the deformation mechanisms of tobermorite, as a simple model system of complex crystalline materials such as C-S-H (the binding phase of concrete) with low Ca/Si ratio. We simulated different loading geometries including conventional loading geometries of tension, compression and shear (global deformation) and nano-indentation loading geometry (local deformation). We found that conventional loading geometries lead to size-independent mechanical properties governed by two key deformation mechanisms: displacive-controlled and diffusive-controlled mechanisms. All shear deformations and the compressive deformations along the [010], “y-”, are dominated by the displacive-controlled mechanisms while all tensile deformations and the compressive deformation along [100] and [001] (“x-”, “z-” respectively), are dominated by the diffusive-controlled deformation mechanism. In general, the structure maintains its structural integrity most in the shear loading, then compressive and finally tensile loading. The atomistic origins of these deformation mechanisms are the relative weak hydrogen-bond connectivity between adjacent silicate layers versus the strong Ca-O and Si-O iono-covalent bonds in the silicate chains^68, 80^.

We found nano-indentation loading geometry results in linear size-dependent indentation modulus and hardness at the nanometer length scales. The size-effect stems from the localized strain gradient (*SG*) in the plastic zone underneath the indenter tip. This *SG* is proportional to the radius of indenter, i.e. *SG∝R*, explaining the linear size-dependent indentation modulus and hardness with respect to the radius of the indenter. The atomistic origin of this *SG* is the local phase transformation of the system represented by the Q^n^ factors (inter-atomic connectivity). The larger the indenter’s radius, the more the transformed Q^n^; especially Q^3^ factors significantly enhance tobermorite’s structural integrity^79^. The evolvement of Q^n^ in the process of nano-indentation loading controls the behavior of the structure. Upon unloading, there are no noticeable changes in the Q^n^ factors, suggesting irreversible local phase transformation and thus permanent (plastic) deformations. Comparing the actual experimental nanoindentation scale and our simulation scale, a transition from size-independent to size-dependent properties occurs. Identification of this critical transition length scale requires future studies.

In brief, our comprehensive investigation of the global and local deformations revealed the three key mechanisms responsible for the mechanical behavior of layered tobermorite: diffusive-controlled deformation mechanism, displacive-controlled deformation mechanism, and strain gradient with local phase transformation. Furthermore, this study classified size-dependent and size-independent loading geometries of layered tobermorite structure, potentially opening up opportunities to use the strong strain gradient at micro-scale as a strengthening method to tune the mechanical performance. To our knowledge, this work is the first report on size-dependent indentation modulus and hardness of layered structures, and can provide useful insights and strategies to probe other complex layered structures such as glassy C-S-H^64^, natural composite structures^81^, and manmade laminated materials.

## Electronic supplementary material

Supplementary information

Supplementary Video S1

Supplementary Video S2

Supplementary Video S3
