# Author Correction: Diffusive, Displacive Deformations and Local Phase Transformation Govern the Mechanics of Layered Crystals: The Case Study of Tobermorite

**DOI:** 10.1038/s41598-018-24596-5

**Published:** 2018-04-16

**Authors:** Lei Tao, Rouzbeh Shahsavari

**Affiliations:** 1 Department of Civil and Environmental Engineering, Rice University, Houston, TX 77005 USA; 2 Department of Materials Science and NanoEngineering, Rice University, Houston, TX 77005 USA; 3 Smalley Institute for Nanoscale Science and Technology, Rice University, Houston, TX 77005 USA

Correction to: *Scientific Reports* 10.1038/s41598-017-05115-4, published online 19 July 2017

This Article contains a typographical error in the Author Contributions section.

“R.S. Designed the research. L.T. carried out the simulations and L.R. and R.S. performed the data analysis and wrote the manuscript.”

should read:

“R.S. Designed the research. L.T. carried out the simulations and L.T. and R.S. performed the data analysis and wrote the manuscript.”

