# Supplementary material for: Diffusive, Displacive Deformations and Local Phase Transformation Govern the Mechanics of Layered Crystals: The Case Study of Tobermorite
Source: Sci Rep. 2017 Jul 19;7:5907. doi: 10.1038/s41598-017-05115-4 (PMC5517551; doi:10.1038/s41598-017-05115-4)
Supplement: Supplementary file 1 — Supplementary information [file 41598_2017_5115_MOESM1_ESM.docx]

**Supplementary Information**

**Diffusive, Displacive Deformations and Local Phase Transformation Govern the Mechanics of Layered Crystals: The Case Study of Tobermorite**

Lei Tao^1^, Rouzbeh Shahsavari^2^*

^1^Department of Civil and Environmental Engineering, Rice University, Houston, TX 77005 USA

^2^Smalley Institute for Nanoscale Science and Technology, Rice University, Houston, TX 77005 USA

*corresponding author: Rouzbeh@rice.edu

**Supplementary information.** Three movies of nano-indentation along X, Y, and Z axis with a 50 Å radius of indenter are provided for reference.

**Video Legends**

**IndentX**. Nano-indentation along X axis with a 50 Angstrom radius of indenter.

**IndentY**. Nano-indentation along Y axis with a 50 Angstrom radius of indenter.

**IndentZ**. Nano-indentation along Z axis with a 50 Angstrom radius of indenter.
